# Clinical Profile of Cyclooxygenase-2 Inhibitors in Treating Non-Small Cell Lung Cancer: A Meta-Analysis of Nine Randomized Clinical Trials

**DOI:** 10.1371/journal.pone.0151939

**Published:** 2016-03-23

**Authors:** Yuan Yuan Zhou, Zhi Gang Hu, Fan Jun Zeng, Jiao Han

**Affiliations:** Department of Respiratory medicine, The first College of Clinical Medicine science, Three Gorges University, Yichang, 443003, People’s Republic of China; Catalan Institute of Oncology, SPAIN

## Abstract

**Background:**

Evidence on the benefits of combining cyclooxygenase-2 inhibitor (COX-2) in treating non-small cell lung cancer (NSCLC) is still controversial. We investigated the efficacy and safety profile of cyclooxygenase-2 inhibitors in treating NSCLC.

**Methods:**

The first meta-analysis of eligible studies was performed to assess the effect of COX-2 inhibitors for patients with NSCLC on the overall response rate (ORR), overall survival (OS), progression-free survival (PFS), one-year survival, and toxicities. The fixed-effects model was used to calculate the pooled RR and HR and between-study heterogeneity was assessed. Subgroup analyses were conducted according to the type of COX-2 inhibitors, treatment pattern, and treatment line.

**Results:**

Nine randomized clinical trials, comprising 1679 patents with NSCLC, were included in the final meta-analysis. The pooled ORR of patients who have NSCLC with COX-2 inhibitors was significantly higher than that without COX-2 inhibitors. In subgroup analysis, significantly increased ORR results were found on celecoxib (RR = 1.29, 95% CI: 1.09, 1.51), rofecoxib (RR = 1.61, 95% CI: 1.14, 2.28), chemotherapy (RR = 1.40, 95% CI: 1.20, 1.63), and first-line treatment (RR = 1.39, 95% CI: 1.19, 1.63). However, COX-2 inhibitors had no effect on the one-year survival, OS, and PFS. Increased RR of leucopenia (RR = 1.21, 95% CI: 1.01, 1.45) and thrombocytopenia (RR = 1.36, 95% CI: 1.06, 1.76) suggested that COX-2 inhibitors increased hematologic toxicities (grade ≥ 3) of chemotherapy

**Conclusions:**

COX-2 inhibitors increased ORR of advanced NSCLC and had no impact on survival indices, but it may increase the risk of hematologic toxicities associated with chemotherapy.

## Introduction

Lung cancer is a major cause of death among patients, and non-small cell lung cancer (NSCLC) accounts for more than 80% of all lung cancers over many countries. The average survival time is 6–10 months for patients with advanced NSCLC in performance status 0–2 receiving palliative first-line chemotherapy [1–4]. Numerous clinical trials about anti-epidermal growth factor receptor (EGFR) agents and anti-anaplastic lymphoma kinase (ALK) agents have demonstrated their superiority in terms of overall response rate (ORR), progression-free survival (PFS), or quality of life (QoL) as compared to standard platinum-based chemotherapy in EGFR and ALK positive patients [5,6]. These examples indicated that new prediction biomarkers can contribute to a remarkable enhancement in treatment outcome.

Cyclooxygenase-2 (COX-2), an important rate-limiting enzyme in prostaglandin synthesis, has been reported to affect apoptosis, angiogenesis, and tumor invasiveness [7]. COX-2 over-expression and prostaglandin biosynthesis have been found in multiple epithelial malignancies with poor prognosis, including lung, breast, and colon [8–10]. Approximately 70% of adenocarcinomas (ADCs) in NSCLC have been found with the increase of COX-2 expression [11,12]. Furthermore, COX-2 inhibitors can prevent the growth of human cancer cells and enhance the activity of standard chemotherapeutic agents [13]. The clinical trial from Edelman and his colleagues showed that patients with low COX-2 protein level exhibit better OS compared with patients with moderate to high expression of COX-2 [14]. Moreover, patients with moderate to high COX-2 expression have a longer median survival (11.2 vs. 3.8 months) when receiving celecoxib than those without celecoxib. The benefits from celecoxib can rise with the increased expression of COX-2. However, other studies indicated that adding COX-2 inhibitors does not improve clinical outcomes of biomarker-selected patients with advanced NSCLC [15,16]. To better assess the efficacy and safety profile of COX-2 inhibitors combined with anticancer therapy for patients with NSCLC, the first meta-analysis of data from published randomized controlled trials (RCTs) in this field was performed.

## Materials and Methods

We carried out this research according to the PRISMA recommendations for meta-analyses [17]. We did not register the protocol.

### Search Strategies

The literature search was conducted on the MEDLINE (1986 to July 2015), EMBASE (July 1986 to July 2015), and Cochrane library databases. The authors used the following keywords: “cyclooxygenase-2 inhibitors,” “cyclooxygenase-2,” and “lung cancer.” Only studies that involved NSCLC patients were included. In addition, the references in the indentified studies were also scanned to complete this search.

### Study Selection

Included studies must meet the following criteria: 1) full papers were published as journal articles in English; 2) the RCTs compared the efficacy and safety profile of adding COX-2 inhibitors to systematic therapy only in NSCLC patients; 3) the study included sufficient data about response, survival, and toxicities; 4) the most recently complete report was included while the same investigators reported data resulting from the same patients.

### Data Extraction and Quality Assessment

Two independent investigators evaluated the titles and abstracts of all study reports identified by the literature search. Disagreements were resolved by consensus through a third investigator. The following data were retrieved from each study: first investigator’s name, year of publication, study design, treatment line, study treatment protocols, and type, dosage, and length of COX-2 inhibitors. The types of outcome measures included the overall response rate (ORR), overall survival(OS), progression-free survival (PFS), and one-year survival. Adverse events were graded according to the National Cancer Institute CTC version 2.0. Only the most frequent events of toxicity were analyzed. Methodological quality of the included studies was assessed using the Cochrane Collaboration tool for assessing the risk of bias [18].

### Statistical Analysis

Differences between the experimental group and the placebo groups were assessed by risk ratio (RR) or hazard ratio(HR) with 95% confidence intervals (CIs). The fixed-effects model (Mantel–Haenszel method) was used to calculate the pooled RR because of the low heterogeneity among studies. The possibility of publication bias was estimated by funnel plots. Heterogeneity among studies was evaluated by calculating P value and the I^2^ measure of inconsistency, which was considered significant if P < 0.10 or I^2^ > 50%. All calculations were carried out using Stata software version 12.0 (Stata Corporation, College Station, TX, USA).

## Results

### Study Selection and Characteristics

**Fig 1:**
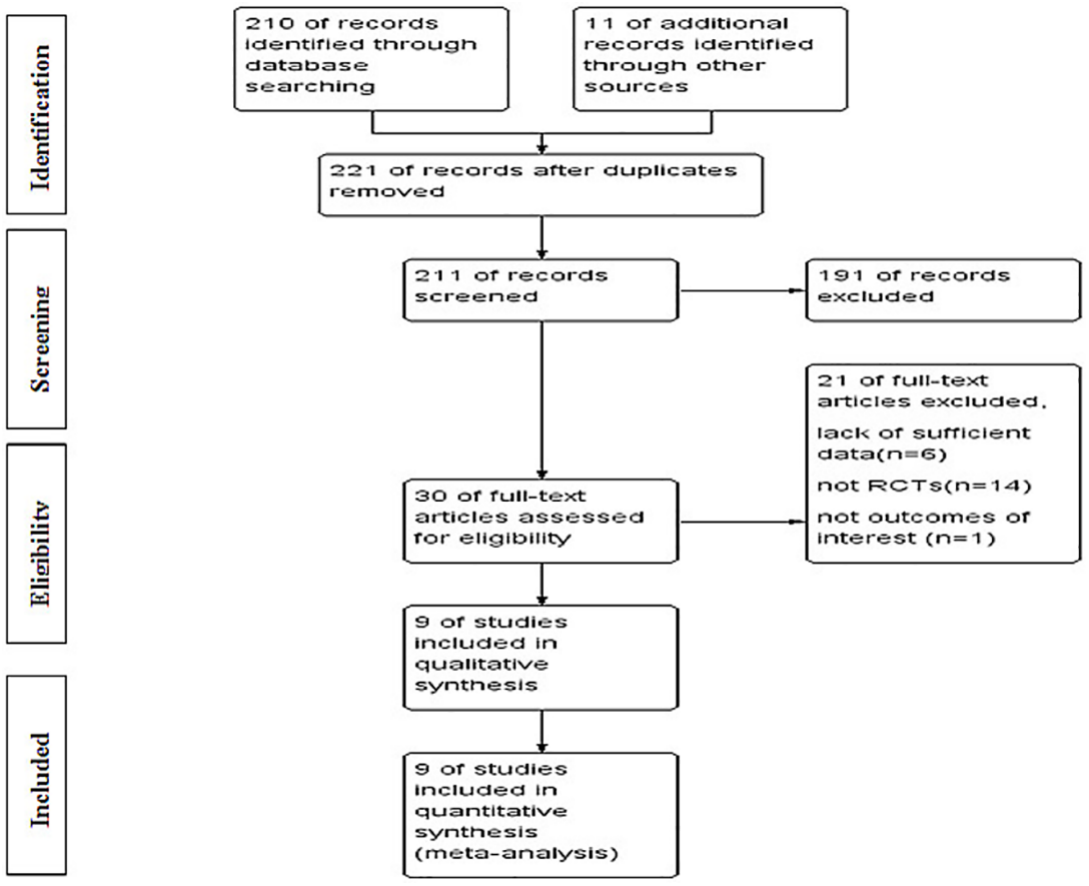
Flow chart indicates the selection of studies. RCT = randomized clinical trial.

**Fig 2:**
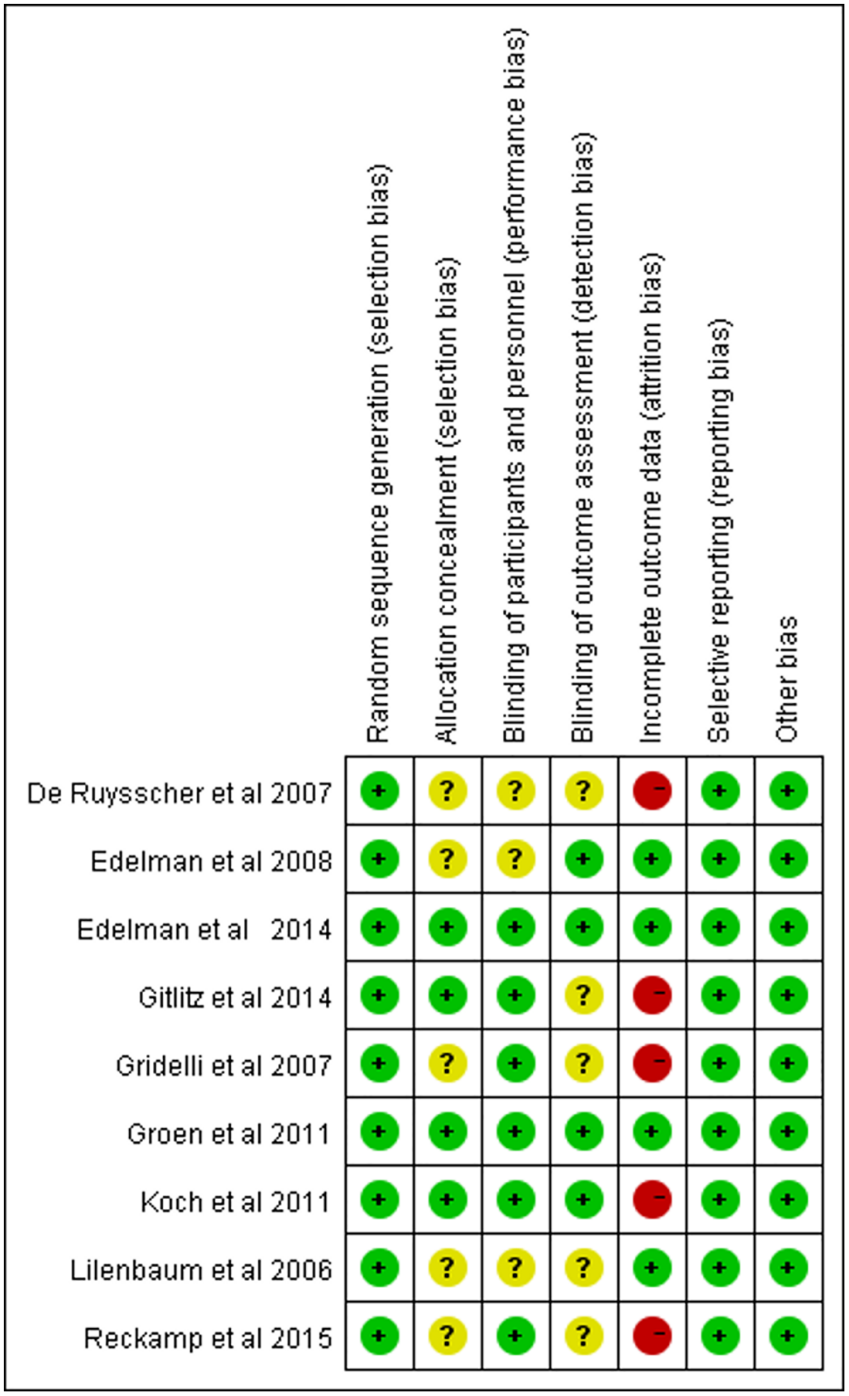
Consensus risk of bias assessments of the included studies. Green: Low risk, Yellow: Unclear, Red: High risk.

Results of the search strategy are shown in Fig 1, and nine studies were included in the study. Fig 2 presents the consensus risk of bias assessments. The eight RCTs involved 1679 patents, ranging from 41 to 561 patients per study [14–16, 19–24]. The major characteristics of the included studies are shown in Table 1. Six studies were Phase II RCTs [14–16, 19, 20] and three were Phase III RCTs [21–23]. Eight studies reported the information of COX-2 inhibitors for treating NSCLC with IIIB or IV stage [14–16,19,21–24]. Nine studies included three COX-2 inhibitors, comprising six studies with celecoxib [14,19–22,24], one with rofecoxib (50 mg qid) [23], and two with apricoxib (400 mg qid) [15,16]. Concomitant treatment included chemotherapy radiotherapy and tyrosine kinase inhibitors (TKIs), which were conducted as first-line treatment [14–16,19,20]. Detailed data are shown in Table 1.

**Table 1:** Characteristics of eligible RCTs included in this meta-analysis.

Study (Year)	Phase	No.Case/ Control	Disease Stage	Treatment Line	Treatment Pattern	Treatment program	Dosage and Length of COX-2 inhibitor
Lilenbaum et al (2006)	II	67/66	IIIB or IV	Second	CT ± Celecoxib	Irinotecan(100 mg/m2) +gemcitabine(1000 mg/m2)/ Irinotecan(60 mg/m2)+ docetaxel (35 mg/m2) day 1, 8	Celecoxib 400 mg, bid, to PD
De Ruysscher et al (2007)	II	21/20	II or III	First	RT ± Celecoxib	Radiotherapy 60 Gy, 2 Gy/d, 5 times /w	Celecoxib 400 mg, bid, 2 y
Gridelli et al (2007)	III	119/121	IIIB or IV	First	CT ± Rofecoxib	Cisplatin (80 mg/m2) day 1 +gemcitabine (1200 mg/m2) day1, 8	Rofecoxib 50 mg/d to PD or 6 cycles
Edelman et al (2008)	II	45/44	IIIB or IV	First	CT ± Celecoxib	Carboplatin (AUC 5.5 mg/mL min) day 1 + gemcitabine(1,000 mg/m2) days 1 8+ zileuton (600 mg) qid	Celecoxib 400 mg, bid, to PD or 6 cycles
Groen et al (2011)	III	281/280	IIIB or IV	First	CT ± Celecoxib	Carboplatin (AUC 6.0 mg/mL min) day 1 +docetaxel (75 mg/m2) day 1	Celecoxib 400mg,bid to PD and ≤3 y
Koch et al (2011)	III	158/158	IIIB or IV	First	CT ± Celecoxib	Carboplatin/cisplatin+ a third generation drug	Celecoxib 400 mg, bid, 1 y
Edelman et al (2014)	II	36/36	IIIB or IV	Second	CT ± Apricoxib	Docetaxel (75 mg/m2) /pemetrexed (500 mg/m2)	Apricoxib 400 mg, qid, to PD
Gitlitz et al (2014)	II	78/42	IIIB or IV	Second	TKIs± Apricoxib	Erlotinib (150 mg/day)	Apricoxib 400 mg, qid, to PD
Reckamp et al(2015)	II	54/53	IIIB or IV	Second	TKIs± Celecoxib	Erlotinib (150 mg/day)	Celecoxib 600 mg, bid, to PD

AUC = area under the curve;

CT = chemotherapy;

PD = progression disease;

RCT = randomized clinical trial;

RT = radiotherapy;

TKIs = tyrosine kinase inhibitors.

### ORRs

**Fig 3:**
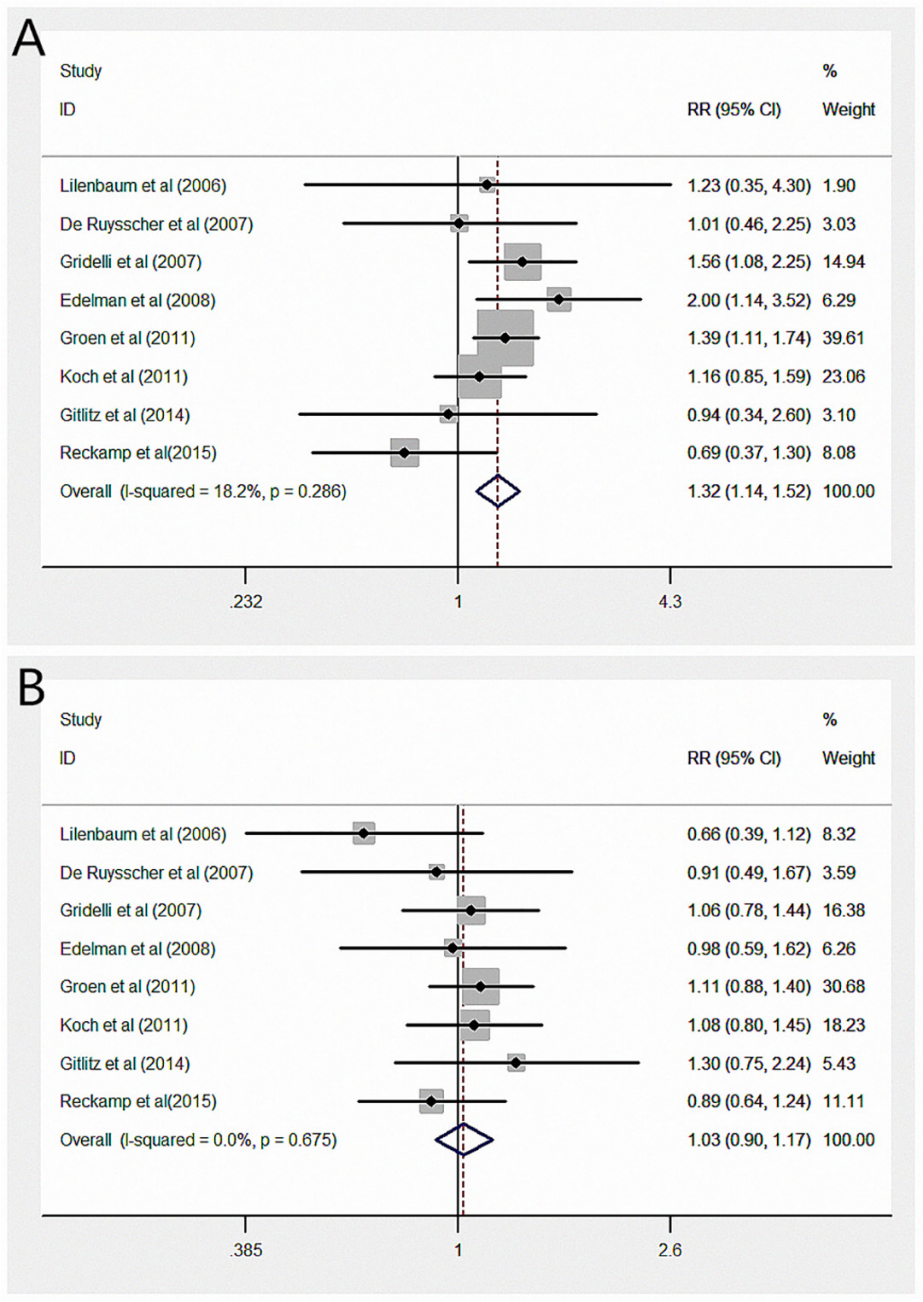
Forest plot of the (A) ORR and (B) one-year survival in patients with NSCLC randomly assigned to COX-2 inhibitors treatment versus placebo/no intervention. ORR = overall response rate.

Eight RCTs reported ORRs [14, 15, 19–24]. The pooled ORR of NSCLC patients with COX-2 inhibitors added to their treatment was 34.1% (264/775), whereas the ORR of patients without added COX-2 inhibitors was 28.2% (208/738). A significant difference of ORR was found between COX-2 inhibitors and placebo. COX-2 inhibitors could significantly improve the ORR of concomitant treatment for advanced NSCLC (RR = 1.32, 95% CI: 1.14, 1.52; Fig 3).

To better assess the efficacy of COX-2 inhibitors for NSCLC, we conducted three subgroup analyses according to types of COX-2 inhibitors (celecoxib, rofecoxib, or apricoxib), treatment pattern (with chemotherapy, radiotherapy or TKIs), and treatment line (first or second). When grouped by types of COX-2 inhibitors, the combined RR was 1.29 (95% CI: 1.09, 1.51) for celecoxib, 1.61 (95% CI: 1.14, 2.28) for rofecoxib, and 0.94 (95% CI: 0.34, 2.60) for apricoxib. We found a statistically significant effect of COX-2 inhibitors added to first-line treatment for advanced NSCLC (RR = 1.39, 95% CI: 1.19, 1.63). Significantly increased ORR was also observed in COX-2 inhibitors with chemotherapy (RR = 1.40, 95% CI: 1.20, 1.63). No apparent heterogeneity was noted among the studies. Detailed data are shown in Table 2.

**Table 2:** Main ORR and survival results extracted from the included RCTs.

	Experimental				Placebo/no Intervention			
Study (Year)	ORR	PFS mo	OS mo	1-y Survival	ORR	PFS mo	OS mo	1-y Survival
Lilenbaum et al (2006)	9.80%	1.8	6.3	23.88%	8.00%	2.1	9	36.36%
De Ruysscher et al (2007)	46.67%	NA	24.2	50.00%	46.15%	NA	15.9	55.00%
Gridelli et al (2007)	41.18%	NA	10.3	42.02%	26.45%	NA	10.3	39.67%
Edelman et al (2008)	24.44%	6.5	9.4	NA	25.00%	4.2	9.4	NA
Groen et al (2011)	41.64%	4.5	8.2	35.23%	30.00%	4	8.2	31.79%
Koch et al (2011)	36.08%	6.1	8.9	36.08%	31.01%	6.5	7.9	33.54%
Edelman et al (2014)	NA	2.8	7.8	NA	NA	3.2	9.6	NA
Gitlitz et al (2014)	12.00%	NA	7.4	NA	12.82%	NA	6.4	NA
Reckamp et al(2015)	22.64%	5.4	12.9	53.70%	32.69%	3.5	14	60.38%

CR = complete release;

NR = not reported;

ORR = over all response rate;

OS = over all survival;

PD = progress disease;

PFS = progression-free survival;

PR = partial release;

RCT = randomized clinical trial;

SD = stable disease.

ORR = (CR + PR)/(SD +PD).;

1-y Mortality = No. alive /No. dead.

### Survival Indices

All studies reported OS durations [14–16,19–24]. Only four studies provided available data to calculate pooled HR [15,21–23]. The pooled HR indicated that the difference of OS durations of patients between study arm and control arm was not statistically significant(HR = 0.97, 95% CI:0.83, 1.14). Seven studies reported PFS durations [14, 15, 19, 21–24]. Five studies provided available data to calculate pooled HR[15, 21–24] The pooled HR suggested that PFS durations of patients treated with or without COX-2 inhibitors had no statistical difference (HR = 0.93, 95% CI:0.81, 1.07).

Eight of the RCTs reported one-year survival rates[14, 15, 19–23]. The one-year survival rate for patients with COX-2 inhibitors did not significantly decrease compared with that for patients without COX-2 inhibitors (RR = 1.03, 95% CI: 0.90, 1.17; Fig 3). As previously mentioned, we also created three subgroup analyses to detect the potential benefit of COX-2 inhibitors for treatment of advanced NSCLC patients. Unfortunately, no clinical profit in one-year survival was found among the groups. A random-effects model was used to evaluate the effect of COX-2 inhibitors with second-line treatment because of apparent heterogeneity. However, the final results remained the same and indicated no statistical significance. Detailed data are shown in Table 3.

**Table 3:** Meta-analysis of ORR and one-year Survival in subgroups on the basis of Cox-2 inhibitor, treatment line, and treatment protocol.

	ORR			one-year Survival		
	N	RR (95%)	Heterogeneity (I2, P)	N	RR (95%)	Heterogeneity (I2, P)
Cox-2 inhibitor type						
Celecoxib	6	1.29 (1.09, 1.51)	30.8%, 0.205	6	1.00 (0.87,1.16)	0%, 0.557
Rofecoxib	1	1.56 (1.08, 2.25)	—	1	1.06 (0.78, 1.44)	—
Apricoxib	1	0.94 (0.34, 2.60)	—	1	1.30 (0.75, 2.24)	—
Treatment line						
Frist	5	1.39 (1.19, 1.63)	0%, 0.430	5	1.07 (0.92, 1.24)	0%, 0.975
Secord	3	0.83 (0.51, 1.36)	0%, 0.692	3	0.90 (0.70, 1.16)	35.2%, 0.214
Treatment protocol						
CT±Cox-2 inhibitor	5	1.40 (1.20, 1.63)	0%, 0.515	5	1.03 (0.89, 1.19)	0%, 0.516
RT±Cox-2 inhibitor	1	1.01 (0.46, 2.25)	—	1	0.91 (0.49, 1.67)	—
TKIs±Cox-2 inhibitor	2	0.76 (0.44, 1.30)	0%, 0.626	2	1.02 (0.77, 1.370)	30.3%, 0.231

CT = chemotherapy;

N = number of included studies;

ORR = overall response rate;

RR = risk ratio;

RT = radiotherapy;— = cannot be calculated.

### QoL

Four studies reported QoL [19, 21–23], which was mainly estimated by the European Organization for Research and Treatment of Cancer Core Quality-of-Life Questionnaire C30 (QLQ-C30), expect for one study [19]. No significant score differences were found between the study groups and the placebo groups in all studies. However, as expected, the use of COX-2 inhibitors could decrease the pain score of the patients with advanced NSCLC [19, 22, 23]. In addition, rofecoxib was reported to improve sleeping, fatigue, physical, and emotional and role functioning of NSCLC patients [23].

### Toxicities

We analyzed common toxicities and some toxicities caused by COX-2 inhibitors, which were reported in more than two studies. These toxicities included hematological events (amenia, leucopenia, neutropenia, and thrombocytopenia), gastrointestinal events (diarrhea, nausea/vomiting), fatigue, thrombosis or embolism, cardiac ischemia, dyspnea, and allergy. Each toxicity was divided into two groups according to the National Cancer Institute Common Toxicity Criteria (version 2) in experimental arm, namely, one group (grade ≥ 3) and the other group (grade < 3). The combined RR of leucopenia and thrombocytopenia was 1.21 (95% CI: 1.01, 1.45) and 1.36 (95% CI: 1.06, 1.76), respectively, suggesting that COX-2 inhibitors increased hematologic toxicities (grade ≥ 3) related to chemotherapy. COX-2 inhibitors for treating NSCLC did not increase the risk of thrombosis or embolism (RR = 1.23; 95% CI: 0.71, 2.14) and the risk of cardiac ischemia (RR = 2.35; 95% CI: 0.61, 9.0). Significantly increased risks of other toxicities were not found. Detailed data are shown in Table 4. In addition, only four studies had a clear description of grade 5 adverse events (toxic death) [14, 16, 22, 23]. Two studies each reported a myocardial infarction in control arm [14, 22]. Another study suggested that control arm had more toxic deaths (6 vs 1) than study arm [23]. The study of Edelman and his colleagues reported one colon perforation in study arm [16].

**Table 4:** Meta-analysis of the toxicities in patients with cancer randomly assigned to celecoxib or placebo/no intervention.

Toxicity	N	Experiment	Placebo	RR (95% CI)	Heterogeneity
		(No. Grade≥3/Other)			(I2,P)
Hematology					
Hemoglobin	6	39/461	35/428	1.05 (0.68, 1.60)	11.9%, 0.339
Leucopenia	5	176/416	145/448	1.21 (1.01,1.45)	32.4%, 0.218
Neutropenia	4	200/346	189/357	1.11 (0.96,1.30)	0.0%, 0.366
Platelets	6	111/592	81/620	1.36 (1.06,1.76)	0.0%, 0.597
Gastrointestology					
Nausea/vomiting	5	27/530	25/497	1.06 (0.62,1.79)	36.9%, 0.175
Diarrhoea	4	21/523	13/495	1.44 (0.73,2.85)	24.4%, 0.265
Fatigue	5	22/558	33/511	0.64 (0.38,1.08)	0.0%, 0.564
Thrombosis or embolism	5	26/611	21/617	1.23 (0.71, 2.14)	0.0%, 0.779
Cardiac ischaemia	3	6/314	2/317	2.35 (0.61, 9.0)	13.4%, 0.315
Dyspnea	3	11/384	5/353	1.61 (0.62,4.20)	9.9%, 0.329
Allergy	3	8/427	9/427	0.89 (0.36,2.22)	0.0%, 0.423

N = number of included studies;

RR = relative risk.

### Sensitivity Analysis and Publication Bias

**Fig 4:**
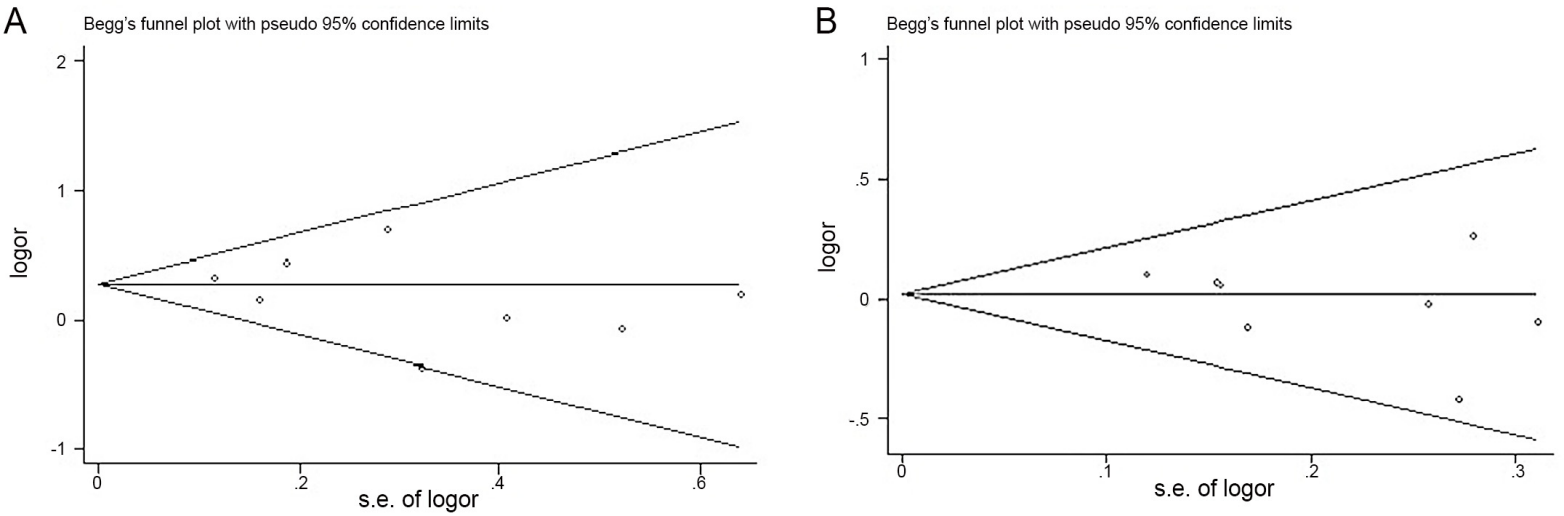
Funnel plot of risk ratio for studies included in the meta-analysis. analysis. (A)ORR, P = 0.43, Egger’s test; (B) one-year survival, P = 0.297, Egger’s test. ORR = overall response rate.

A fixed-effects model was used to assess sensitivity. When we respectively removed the study of the smallest sample size or the study of the largest sample size, the results of meta-analysis did not significantly change compared with the results of the primary analysis. When we removed the study of the smallest sample size, the pooled RR was 1.32(95% CI: 1.14, 1.54) in ORR and 1.03(95% CI: 0.91, 1.18) in one-year survival. When we removed the study of the largest smallest sample size, the pooled RR was 1.27(95% CI: 1.04, 1.54) in ORR and 0.99(95% CI: 0.85, 1.15) in one-year survival. Begg’s funnel plot and Egger’s test were used to assess the publication bias of the included RCTs. Begg’s funnel plot of RRs did not find asymmetry, and evaluation with Egger’s test indicated no significant publication bias (P > 0.05; Fig 4).

## Discussion

COX-2 is up-regulated in response to various substances, including growth factors, cytokines, and carcinogens. Increased COX-2 and prostaglandin E levels have been implicated in tumor invasion, angiogenesis, suppression of antitumor immunity, and resistance to apoptosis [25]. A newly published meta-analysis implied that the over-expression of COX-2 is associated with poor survival and prognosis in lung cancer patients, especially ADC and Stage I NSCLC [26]. Celecoxib, a highly selective COX-2 inhibitor, is often used to study the anti-neoplastic activity for lung cancer cell and lung cancer. Celecoxib was observed to induce lung cancer cell apoptosis by the intrinsic and extrinsic apoptosis pathways, including mitochondrial apoptosis pathway and FADD- and caspase-8-dependent death mechanism [27]. A review indicated that the use of celecoxib may be of specific value for treating apoptosis-resistant tumors with overexpression of Mcl-1 or Bcl-2 [27]. In addition, COX-2 inhibitors may reduce the adverse events caused by radiotherapy and chemotherapy, such as radiation pneumonia [20] and diarrhea [15]. However, clinical trials implied that COX-2 inhibitors do not always improve ORR and survival indices of patients with NSCLC, but they shorten the OS and PFS [19]. Therefore, quantitative assessment of the clinical profile of COX-2 inhibitors for NSCLC patients is necessary.

To the best of our knowledge, this meta-analysis is the first to evaluate the clinical profile and toxicities of COX-2 inhibitors for treating advanced NSCLC. This present meta-analysis combined nine published RCTs including 1679 NSCLC patients to yield summary statistics. The results demonstrated that COX-2 inhibitors might apparently increase the ORR in the advanced NSCLC patients. In subgroup analysis, we observed that celecoxib and rofecoxib might provide higher ORR than placebo arms. When grouped by treatment line, COX-2 inhibitors combined into first-line treatment showed a significant effect in ORR compared with the control arms. However, increased ORR was not observed in second-line treatment with COX-2 inhibitors. Based on treatment pattern, we observed a statistically significant favorable effect of chemotherapy with COX-2 inhibitors in ORR but no change in radiotherapy or TKIs with COX-2 inhibitors. Similar results were not obtained in one-year survival. In all subgroup analyses, no significant differences in one-year survival were found between the study groups and placebo groups. In addition, COX-2 inhibitors had no significant influence on OS and PFS. Although COX-2 inhibitors did not significantly reduce the score of QLQ-C30, the improvement in pain was reported in three studies [19,22,23]. These results suggested that first-line chemotherapy with COX-2 inhibitors for advanced NSCLC patients may obtain a higher ORR compared with other combined treatment options. Indeed, some studies demonstrated that COX-2 inhibitors could enhance antitumor activity of conventional anticancer agents in vitro and in vivo, especially taxanes [13,28]. Our study also proved that COX-2 inhibitors combined with first-line chemotherapy could gain better treatment response. However, we did not find that first-line chemotherapy with COX-2 inhibitors improved survival indices for advanced NSCLC patients. A potential explanation is that COX-2 inhibitor could reduce the intratumoral levels of COX-2 and prostaglandin M (PGE-M), which high expression was caused by chemotherapy [28]. In the study of Mutter et al, there was an explicit association between PGE-M levels with response (*P* = 0.005) but not with survival (*P* = 0.114) [29]. Thus, we deemed that COX-2 inhibitions may contribute to local control by improving the effects of chemotherapy and have less or no impact on survival indices. In addition, some factors were described to enhance the efficacy of COX-2 inhibitors for treating advanced NSCLC. One study indicated that median OS of patients (≤65 years) was 12.2 months in the study arm compared with 4.0 months in the placebo group [15]. Another two papers implied that the median OS with COX-2 inhibitors was longer than that with placebo in female patients [14, 22]. When the index of expression of COX-2 was more than 4, the patients with celecoxib had better OS and PFS than those without celecoxib [14]. If pretreatment plasma levels of vascular endothelial growth factor (VEGF) were restricted to lower than 200 pg/ml, celecoxib had a protective effect on survival compared with placebo [30].

Toxicities, especially cardiovascular toxicity, induced by COX-2 inhibitors limit its applications and research for cancer. In particular, the Adenomatous Polyp Prevention on Vioxx Trial suggested that rofecoxib may accelerate the risk of thrombotic events, mainly myocardial infarctions and ischemic cerebrovascular events [31]. Therefore, two RCTs did not complete the recruitment of volunteers according to the original plan [20,23]. A newly published meta-analysis indicated that long-term use of celecoxib for treating advanced cancers may significantly raise the risk of grade 3 and grade 4 cardiovascular events (RR = 1.78; 95% CI: 1.30–2.43) [32]. In the present meta-analysis, we did not find that COX-2 inhibitors for treating NSCLC could expand the risk of thrombosis or embolism (RR = 1.23; 95% CI: 0.71, 2.14) and the risk of cardiac ischemia (RR = 2.35; 95% CI: 0.61, 9.0). However, the risk of leucopenia and thrombocytopenia in the experiment arms was notable because of the apparent increase in RR (see Table 4). One study implied that COX-2 may play an important role in the recovery of the bone marrow after chemotherapy [33], which is a possible explanation for a higher frequency of leucopenia and thrombocytopenia in the experiment arms. In addition, apricoxib can effectively reduce the risk of diarrhea caused by erlotinib.

Despite no significant heterogeneity in publication bias, our meta-analysis also had some limitations. First, most patients in our meta-analysis were in stage IIIB or IV of NSCLC [14–16,19,21–24] and only one study with stage II-III NSCLC[20], so we could not evaluate the efficacy of COX-2 inhibitors for early NSCLC. Second, the meta-analysis was possibly influenced by the poor recruitment in two RCTs [19, 23]. Third, not all RCTs provided sufficient data with respect to ORR and survival indices, which affected the pooled results in the present meta-analysis. Furthermore, only patients with a ≥50% decrease in urinary PGE-M after 5 days of treatment with apricoxib could enroll in two studies [15,16]. In addition, only apricoxib combined with second-line treatment was reported. Therefore, the results of apricoxib for NSCLC would greatly suffer because of selection bias. Finally, there were three phase III trials and six phase II trials in this meta-analysis. Only one study with stage II-III NSCLC treated with radiotherapy with or without concurrent celecoxib was included this meta-analysis. These factors indicate that our study maybe have clinical and methodological heterogeneity.

## Conclusions

This meta-analysis suggested that COX-2 inhibitors may increase ORR of chemotherapy with advanced NSCLC, especially combined with first-line treatment. However, no similar change was found in the survival indices. In addition, COX-2 inhibitors may enlarge myelotoxicity induced by chemotherapy. Despite no significant extension in cardiovascular toxicity, the use of COX-2 inhibitors is prudent for patients with a history of cardiac diseases. Based on these findings, benefits versus hazards of COX-2 inhibitors for treating advanced NSCLC need to be carefully considered.

## Supporting Information

S1 PRISMA Checklist PRISMA 2009 checklist. (DOC)

## Abbreviations

ADC: adenocarcinomas
ALK: anaplastic lymphoma kinase
CI: confidence intervals
COX-2: cyclooxygenase-2
EGFR: epidermal growth factor receptor
NSCLC: non-small cell lung cancer
ORR: overall response rate
OS: overall survival
PFS: progression-free survival
RCTs: randomized controlled trials
TKIs: tyrosine kinase inhibitors
VEGF: vascular endothelial growth factor
