# Supplementary material for: Clinical Profile of Cyclooxygenase-2 Inhibitors in Treating Non-Small Cell Lung Cancer: A Meta-Analysis of Nine Randomized Clinical Trials
Source: PLoS One. 2016 Mar 23;11(3):e0151939. doi: 10.1371/journal.pone.0151939 (PMC4805232; doi:10.1371/journal.pone.0151939)
Supplement: S1 PRISMA Checklist — (DOC) [file pone.0151939.s001.doc]

| **Section/topic 项目** | **#编号** | **Checklist item 条目清单** | **Reported on page #** |
| --- | --- | --- | --- |
| **TITLE 标题** | | |  |
| Title 标题 | √1 | Identify the report as a systematic review, meta-analysis, or both.  明确本研究报告是系统综述、meta分析．还是两者兼有 | 1/7 |
| **ABSTRACT 摘要** | | |  |
| Structured summary  结构式摘要 | √2 | Provide a structured summary including, as applicable: background; objectives; data sources; study eligibility criteria, participants, and interventions; study appraisal and synthesis methods; results; limitations; conclusions and implications of key findings; systematic review registration number.  提供结构式摘要，包括背景、目的、资料来源、纳入研究的标准、研究对象和下预措施、研究评价和综  合的方法、结果、局限性，结论和主要发现、系统综述的注册号 | 1/7-8 |
| **INTRODUCTION 前言** | | |  |
| Rationale 理论基础 | √3 | Describe the rationale for the review in the context of what is already known.  介绍当前已知的研究理论基础 | 8 |
| Objectives 目的 | √4 | Provide an explicit statement of questions being addressed with reference to participants, interventions, comparisons, outcomes, and study design (PICOS).  通过对研究对象、干预措施、对照措施、结局指标和研究类型(participants，interventions，comparisons，outcomes，study design，P1C()s)5个方面为导向的问题提出所需要解决的清晰明确的研究问题 | 8 |
| **METHODS 方法** | | |  |
| Protocol and registration  方案和注册 | √5 | Indicate if a review protocol exists, if and where it can be accessed (e.g., Web address), and, if available, provide registration information including registration number.  如果已有研究方案，则说明方案内容并给出可获得该方案的途径(如网址)，并且提供现有的已注  册的研究信息，包括注册号 | 9 |
| Eligibility criteria  纳入标准 | √6 | Specify study characteristics (e.g., PICOS, length of follow-up) and report characteristics (e.g., years considered, language, publication status) used as criteria for eligibility, giving rationale.  将指定的研究特征(如PICOS和随访的期限)和报告的特征(如检索年限、语种和发表情况)作为  纳入研究的标准．并给出合理的说明 | 9 |
| Information sources  信息来源 | √7 | Describe all information sources (e.g., databases with dates of coverage, contact with study authors to identify additional studies) in the search and date last searched.  针对每次检索及最终检索的结果描述所有文献信息的来源(如资料库文献，与研究作者联系获取  相应的文献) | 9 |
| Search  检索 | √8 | Present full electronic search strategy for at least one database, including any limits used, such that it could be repeated.  至少说明一个资料库的检索方法。包含所有的检索策略的使用，使得检索结果可以重现 | 9 |
| Study selection  研究选择 | √9 | State the process for selecting studies (i.e., screening, eligibility, included in systematic review, and, if applicable, included in the meta-analysis).  说明纳入研究被选择的过程(包括初筛、合格性鉴定及纳入系统综述等步骤，据实还可包括纳入meta分析的过程) | 9 |
| Data collection process  资料提取 | √10 | Describe method of data extraction from reports (e.g., piloted forms, independently, in duplicate) and any processes for obtaining and confirming data from investigators.  描述资料提取的方法(例如顶提取表格、独立提取、重复提取)以及任何向报告作者获取或确认资料的过程 | 9 |
| Data items  资料条目 | √11 | List and define all variables for which data were sought (e.g., PICOS, funding sources) and any assumptions and simplifications made.  列出并说明所有资料相关的条目(如PICOS和资金来源)，以及作出的任何推断和简化形式 | 9 |
| Risk of bias in individual studies  单个研究存在的偏倚 | √12 | Describe methods used for assessing risk of bias of individual studies (including specification of whether this was done at the study or outcome level), and how this information is to be used in any data synthesis.  描述用于评价单个研究偏倚的方法(包括该方法是否用于研究层面或结局层面)，以及在资料综合  中该信息如何被利用 | 9 |
| Summary measures  概括效应指标 | √13 | State the principal summary measures (e.g., risk ratio, difference in means).  说明主要的综合结局指标，如危险度比值(risk ratio)、均值差(difference in means) | 9 |
| Synthesis of results  结果综合 | √14 | Describe the methods of handling data and combining results of studies, if done, including measures of consistency (e.g., I^2^) for each meta-analysis.  描述结果综合的方法，如果进行了meta分析。则说明异质性检验的方法 | 9 |

Page 1 of 2

| Risk of bias across studies  研究偏倚 | √15 | Specify any assessment of risk of bias that may affect the cumulative evidence (e.g., publication bias, selective reporting within studies).  详细评估可能影响数据综合结果的可能存在的偏倚(如发表偏倚和研究中的选择性报告偏倚) | 9 |
| --- | --- | --- | --- |
| Additional analyses  其他分析 | √16 | Describe methods of additional analyses (e.g., sensitivity or subgroup analyses, meta-regression), if done, indicating which were pre-specified.  对研究中其他的分析方法进行描述(如敏感性分析或亚组分析，meta回归分析)，并说明哪些分析是预先制定的 | 9 |
| **RESULTS 结果** | | |  |
| Study selection  研究选择 | √17 | Give numbers of studies screened, assessed for eligibility, and included in the review, with reasons for exclusions at each stage, ideally with a flow diagram.  报告初筛的文献数，评价符合纳入标准的文献数以及最终纳入研究的文献数。同时给出每一步排  除文献的原因，最好提供漉程图 | 9-10 |
| Study characteristics  研究特征 | √18 | For each study, present characteristics for which data were extracted (e.g., study size, PICOS, follow-up period) and provide the citations.  说明每一个被提取资料的文献的特征(如样本含量、PICOS和随访时间)并提供引文出处 | 10 |
| Risk of bias within studies  研究内部偏倚风险 | √19 | Present data on risk of bias of each study and, if available, any outcome level assessment (see item 12).  说明每个研究中可能存在偏倚的相关数据。如果条件允许，还需要说明结局层面的评估(见条目12) | 10 |
| Results of individual studies  单个研究的结果 | √20 | For all outcomes considered (benefits or harms), present, for each study: (a) simple summary data for each intervention group (b) effect estimates and confidence intervals, ideally with a forest plot.  针对所有结局指标(有效性或有害性)。说明每个研究的各干预组结果的简单合并(a)。以及综合效应值及其可信区间(b)。最好以森林图形式报告 | 10 |
| Synthesis of results  结果的综合 | √21 | Present results of each meta-analysis done, including confidence intervals and measures of consistency.  说明每个meta分析的结果，包括可信区间和异质惟检验的结果 | 10-11 |
| Risk of bias across studies  研究间偏倚 | √22 | Present results of any assessment of risk of bias across studies (see Item 15).  说明研究间可能存在偏倚的评价结果(见条目15) | 11 |
| Additional analysis  其他分析 | √23 | Give results of additional analyses, if done (e.g., sensitivity or subgroup analyses, meta-regression [see Item 16]).  如果有，给出其他分析的结果(如敏感性分析或哑组分析．meta一回归分析，见条目16) | 11 |
| **DISCUSSION 讨论** | | |  |
| Summary of evidence  证据总结 | √24 | Summarize the main findings including the strength of evidence for each main outcome; consider their relevance to key groups (e.g., healthcare providers, users, and policy makers).  总结研究的主要发现，包括每一个主要结局的证据强度；分析它们与主要利益集团的关联性(如医疗保健的提供者、使用者及政策决策者) | 11-12 |
| Limitations  局限性 | √25 | Discuss limitations at study and outcome level (e.g., risk of bias), and at review-level (e.g., incomplete retrieval of identified research, reporting bias).  探讨研究层面和结局层面的局限性(如偏倚的风险)，以及系统综述的局限性(如检索不全面，报告  偏倚等) | 12 |
| Conclusions | √26 | Provide a general interpretation of the results in the context of other evidence, and implications for future research.  给出对结果的概要性的解析，并提出对未来研究的提示 | 13 |
| **FUNDING资金支持** | | |  |
| Funding  资金 | √27 | Describe sources of funding for the systematic review and other support (e.g., supply of data); role of funders for the systematic review.及资助者在完成系统综述巾所起的  描述本系统综述的资金来源和其他支持(如提供资料)以作用 | 1 |

*From:*  Moher D, Liberati A, Tetzlaff J, Altman DG, The PRISMA Group (2009). Preferred Reporting Items for Systematic Reviews and Meta-Analyses: The PRISMA Statement. PLoS Med 6(6): e1000097. doi:10.1371/journal.pmed1000097

For more information, visit: **www.prisma-statement.org**.

**My manuscript contains all the checklist above.**
